# The Patterns of Migration of Potentially Toxic Elements from Coal Mining Subsidence Areas and Associated Soils to Waterlogged Areas

**DOI:** 10.3390/toxics11110888

**Published:** 2023-10-30

**Authors:** Min Tan, Jihong Dong, Junfeng Qu, Ming Hao

**Affiliations:** 1 School of Public Policy and Management, China University of Mining and Technology, Xuzhou 221116, China; db16160031b0@cumt.edu.cn; 2 School of Environment and Spatial Informatics, China University of Mining and Technology, Xuzhou 221116, China; haoming@cumt.edu.cn; 3 Carbon Neutrality Institute, China University of Mining and Technology, Xuzhou 221008, China; qjf4209779@163.com; 4 Xuzhou Institute of Ecological Civilization Construction, Xuzhou 221008, China

**Keywords:** potentially toxic elements, migration pattern, landscaped wetland, fish farming, fish–photovoltaic complementary wetlands, photovoltaic wetland

## Abstract

It is crucial for effectively controlling potentially toxic element (PTE) pollution to understand the pollution situation, ecological risks, health risks, and migration patterns of PTEs. However, currently, no research has been conducted on the migration patterns of soil PTEs from coal mining subsidence areas to waterlogged areas under different restoration modes. In this study, a total of 15 sediment samples and 60 soil samples were collected from landscaped wetlands, aquaculture wetland, fish–photovoltaic complementary wetland, photovoltaic wetland, and waterlogged areas with untreated coal mining subsidence. The PTE pollution status, ecological risks, health risks, migration patterns, and the important factors influencing the migration were analyzed. The results indicated that the comprehensive pollution level of PTEs in waterlogged areas with coal mining subsidence can be reduced by developing them into landscaped wetlands, aquaculture wetlands, fish–photovoltaic complementary wetlands, and photovoltaic wetlands. Additionally, the closer to the waterlogged area, the higher the Cu content in the subsidence area soil is, reaching its peak in the waterlogged area. The Cd was influenced positively by SOC and pH. The research results were of great significance for formulating reclamation plans for waterlogged areas and controlling PTE pollution.

## 1. Introduction

Long-term underground coal mining has brought about a series of ecological problems, restraining the sustainable development of society [1]. The most prominent among them is the issue of coal-mining-related subsidence and water accumulation [2,3]. The scale of high-groundwater-level waterlogged areas with coal mining subsidence in China reaches up to 2000 km^2^. The water bodies in the subsided areas are generally closed or semi-closed systems with poor water circulation and limited self-purification capacity, resulting in the disruption of surface water systems and degradation of water quality [4]. Contaminated subsidence water further enters the agricultural system through activities like irrigation [5]. The subsidence water has a high content of potentially toxic elements (PTEs), including metals with a density greater than 4.5 g cm^−3^, such as cadmium (Cd), lead (Pb), chromium (Cr), copper (Cu), zinc (Zn), and mercury (Hg), as well as arsenic (As) and other metalloids [6,7]. PTEs in the polluted irrigation water accumulate in the soil or vegetation influenced by the soil’s cultivated and physiochemical condition (pH, Eh et al.), severely affecting local food security, ecological safety, and social stability [8,9,10]. Restoration is urgently needed for waterlogged areas with coal mining subsidence.

To address the environmental issues in waterlogged areas with coal mining subsidence and make the best use of natural resources, large-scale restoration efforts are currently underway [11,12]. This includes activities such as aquaculture development and the creation of landscaped wetlands within the waterlogged areas [13]. Furthermore, under the pressure of “carbon neutrality” and “carbon emission reduction”, an increasing number of waterlogged areas are being developed as photovoltaic wetlands or fish–photovoltaic complementary wetlands [2,14]. These development approaches can enhance the environmental aesthetics of the waterlogged areas and provide economic and energy value [15,16]. However, it cannot be overlooked that these approaches can introduce additional environmental pressures on aquatic ecosystems [17]. For instance, aquaculture feed is rich in PTEs such as Cu and Zn [18]. Purified urban sewage contains PTEs such as Cd and Cr [19]. Will the introduction of feed or purified sewage into the stagnant water area exacerbate the environmental pressure on the aquatic ecosystem? Do photovoltaic components disrupt the natural equilibrium of the aquatic ecosystem and potentially diminish its self-purification capacity for PTEs? It is crucial for us to investigate the extent of environmental stress that different restoration methods may impose on the aquatic ecosystem.

Furthermore, the main sources of PTEs in soil are coal mining operations, agricultural nonpoint source pollution, transportation, atmospheric deposition, and so on [20,21,22]. As a result, soil PTEs enter the waterlogged areas with coal mining subsidence through surface runoff and agricultural drainage, accumulating in the sediment. Nonpoint source pollution and coal mining pollution exhibit characteristics such as randomness and fuzziness [23], making the study of them challenging. Currently, there is no research conducted on the migration patterns of soil PTEs from coal mining subsidence areas to waterlogged areas.

PTEs have a high toxicity, are difficult to degrade, and can disrupt the ecological balance, posing a threat to human health [24,25,26]. Min Tan [27] and Lingli Zhou [28] found that the contents of Cd, Pb, Cr, Cu, Zn, Hg, and As were relatively high in the mining area environment. It is crucial to understand the pollution levels, ecological risks, health risks, and migration patterns of these seven PTEs in waterlogged areas with coal mining subsidence under different restoration modes. This understanding is vital for ensuring the health of residents living nearby.

For the aforementioned reasons, this study, for the first time, investigated the migration patterns and influencing factors of soil PTEs into subsidence waterlogged areas in a high-groundwater-level coal mining area under different remediation modes. The research aimed to (1) analyze the PTE pollution, ecological risks, and health risks in waterlogged water areas with coal mining subsidence under different remediation modes, (2) investigate the migration patterns of soil PTEs from the surrounding areas of coal mining subsidence to waterlogged areas, and (3) identify the key factors influencing the migration of PTEs. The research findings can provide data support for the management of PTEs and aid in the selection of future management decisions in subsidence areas.

## 2. Materials and Methods

### 2.1. Sample Collection and Analysis

The study area is located in the high-water-table region in eastern China. It has a temperate semi-humid monsoon climate with an average annual temperature of 14.2 °C, an average annual sunshine duration of 2307.9 h, and an average annual precipitation of 816.4 mm. The area is crisscrossed with rivers, and water resources are relatively abundant. The terrain is higher in the southwest and lower in the northeast, forming a typical alluvial plain. The soil is developed from yellow alluvial sediments. The research area has eight mines and is the only coal production base in Jiangsu Province. The proven coal reserves are 2.37 billion tons. Since 1977, a total of 230 million tons of raw coal has been extracted, resulting in significant land subsidence.

**Figure 1:**
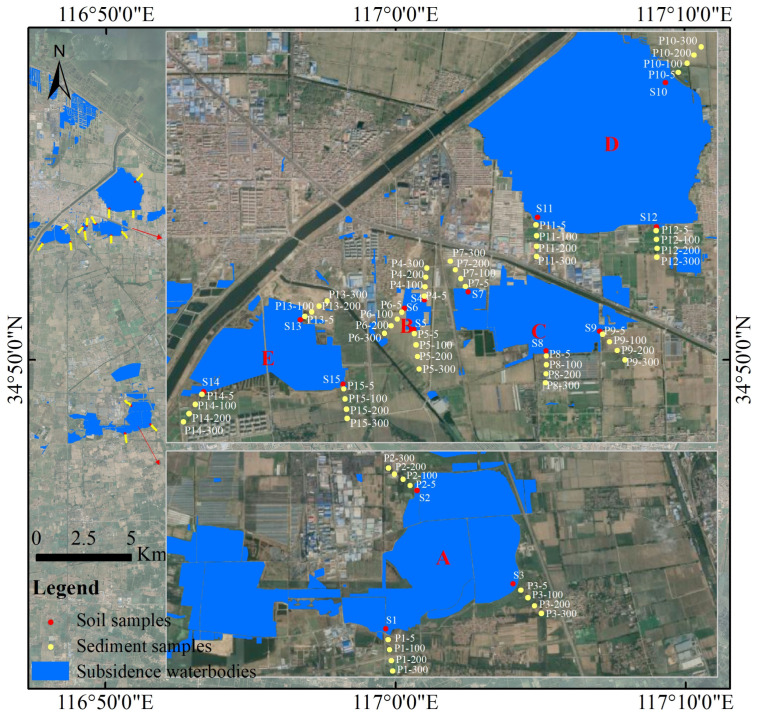
Location of 15 sediment samples and 60 soil samples in the surrounding subsidence waterbodies.

In January 2023, sediment samples were collected from waterlogged areas under five different restoration modes, and soil samples from surrounding farmland in a typical high-water-level coal mining area in China were also collected. The distribution of sampling points is shown in Figure 1. Point A represents Anguo Wetland, a purification-type landscaped wetland that receives treated wastewater from urban sewage treatment plants. Point B represents a waterlogged area used for fish farming. Point C represents a fish–photovoltaic complementary wetland, developed in waterlogged areas with coal mining subsidence. Point D represents a newly developed photovoltaic wetland in 2022. Point E represents a waterlogged area with untreated coal mining subsidence.

Three sample plots were set up in each of the five waterlogged areas, and three sediment samples at a depth of 0–20 cm were randomly collected in each plot. Additionally, surface soil samples (0–20 cm depth) were collected at distances of 5 m, 100 m, 200 m, and 300 m from each sediment plot in the farmland. All samples were cleaned of stones, gravel, and dead leaves, mixed thoroughly, and then divided into two sterile sealed bags. One bag was used for determining the physicochemical indicators, including PTEs, and the other bag was kept as a backup. A total of 15 sediment samples and 60 soil samples were collected in this study. All samples were transported to the laboratory on the day of collection and stored in a dark and cool environment.

Before conducting the analysis, air-dry the soil and sediment samples at room temperature. After removing pebbles and plant residues, sieve the samples using a 2 mm mesh screen, and then store them in polyethylene bags. After the pretreatment, measure the pH value, soil organic carbon (SOC), and indicators of PTEs such as cadmium (Cd), lead (Pb), chromium (Cr), copper (Cu), zinc (Zn), mercury (Hg), and arsenic (As) in the samples. The soil pH was determined using a pH meter (PHBJ-260F, Leici, Shanghai, China), following the reference method of the point-to-point method (NY/T1377-2007) [29]. The determination of SOC (soil organic carbon) was conducted according to the reference method of the potassium dichromate oxidation–spectrophotometric method (HJ 615-2011) for the determination of organic carbon in soil [30]. The instrument used for this analysis was a Total Organic Carbon analyzer (TOC-LCPH, Shimauzu, Kyoto, Japan). For the determination of Cd, Pb, Cr, Cu, Zn, and As in soil and sediment, the reference method used was the aqua regia extraction–inductively coupled plasma mass spectrometry method (HJ 803-2016) using an inductively coupled plasma mass spectrometer (PlasmaQuantMS, Analytik Jena AG, Jena, Germany) [31]. Hg was determined following the reference method of catalytic thermal decomposition–cold vapor atomic absorption spectrometry (HJ 923-2017) for the determination of total mercury in soil and sediment using an automated mercury analyzer (DMA-80, Milestone, Modena, Italy) [32]. The detection limits of Cd, Pb, Cr, Cu, Zn, Hg, and As were 0.01 mg kg^−1^, 0.1 mg kg^−1^, 4.0 mg kg^−1^, 1.0 mg kg^−1^, 0.5 mg kg^−1^, 0.2 mg kg^−1^, and 0.01 mg kg^−1^, respectively. The relative standard deviation (RSD) values of Cd, Pb, Cr, Cu, Zn, As, and Hg were 6.70%, 6.31%, 6.67%, 2.75%, 1.01%, 3%, and 5.47%. The accuracies for the Cd, Pb, Cr, Cu, Zn, As, and Hg are 5.5%, 6.7%, 5.7%, 7.1%, 3.6%, 1.12%, and 3.0%, respectively.

### 2.2. Assessment of Trace Metal Pollution in Sediment

The single-factor index is a measure of the pollution level of soil PTEs, which compares the measured data with standard data [33]. The specific calculation formula is as follows:

(1) CF=CiSi

where *CF* is the single pollution index for PTE *i*, *C_i_* is the measured value of PTE *i*, and *Si* is the standard value of PTE *i*. The pollution level was classified based on the magnitude of the *CF* value: *CF* ≤ 1, no pollution; 1 < *CF* ≤ 2, mild pollution; 2 < *CF* ≤ 3, moderate pollution; 3 < *CF*, severe pollution.

The Pollution Load Index (*PLI*) is used to evaluate the environmental pollution pressure of soil PTEs in the study area [34]. When *PLI* is less than 0, it is taken as 0. When *PLI* ≤ 1, it indicates the absence of environmental pollution. When *PLI* > 1, it indicates the presence of environmental pollution. The specific calculation formula for *PLI* is as follows:

(2) $$PLI={\left(CF_{1}\times CF_{2}\times \cdots CF_{i}\times \cdots \times CF_{n}\right)}^{1/n}$$

where *CF_i_* represents the single-factor pollution index of the PTE.

### 2.3. Sediment Quality Guidelines (SQGs)

The sediment quality guidelines (SQGs) establish threshold values for specific chemical substances in sediments that do not pose a risk of harm to benthic organisms or other relevant water body functions [35]. Referring to the guidelines proposed by Macdonald et al. [35], the threshold effects level (*TEL*), probable effects level (*PEL*), and the biological effects induced by PTEs in this study were evaluated. According to the values, they were divided into three groups: rarely (below *TEL*), occasionally (between *TEL* and *PEL*), and frequently (above *PEL*) [36,37].

### 2.4. Ecological Risk Assessment

The potential ecological risk index (*RI*) is an ecological risk assessment method established by Swedish scientist Hakanson in 1980 [33]. This method takes into account factors such as PTE content and level of toxic pollution. It has been widely applied in the assessment of environmental pollution and ecological risk in sediments, soils, and other areas [33,38]. The specific calculation formula is as follows.

(3) $$E_{r}^{i}=T_{t}^{i}\times CF_{i}$$

(4) $$RI=\sum E_{r}^{i}$$

In the equation, $E_{r}^{i}$ represents the potential ecological risk factor, and $T_{t}^{i}$ represents the toxicity response coefficient of PTE *i*. The coefficients for Cu, Zn, Cr, Cd, Hg, As, and Pb were 5, 1, 2, 30, 40, 10, and 5, respectively [39]. *CF_i_* represents the single-factor pollution index of the PTE, and *RI* represents the potential ecological risk index. The pollution levels corresponding to the $E_{r}^{i}$ and *RI* are shown in Table 1.

### 2.5. Human Health Risk Assessment

The health risks of PTEs to the human body can be broadly categorized into two major types: carcinogenic health risks and noncarcinogenic health risks [40]. The most widely used model for assessing health risks associated with PTEs is the health risk assessment model recommended by the U.S. Environmental Protection Agency (USEPA). The pathways of exposure to PTEs in the environment include ingestion through food and water, dermal contact, and inhalation through respiration.

The health hazards of PTEs to the human body depend on factors such as duration of exposure, route of exposure, and age of the exposed individual [41,42,43]. Based on the methodology proposed by the Risk Assessment Information System (RAIS) in the United States, the health risks for children and adults were assessed. The average daily intake (*ADI*) (mg kg^−1^ d^−1^) was calculated using the following formula:

(5) $$ADI=\frac{C\times EF\times ED\times IngR\times RBA}{AT\times BW}\times 10^{-6}$$

where *c* is the PTE content, mg kg^−1^, and *EF* represents the annual average exposure days, d a^−1^. *ED* represents the exposure years, calculated separately for children and adults as 6a and 26a, respectively. *IngR* is the ingestion rate, calculated separately for children and adults as 200 mg d^−1^ and 100 mg d^−1^, respectively. *RBA* represents the relative bioavailability, with an As value of 0.6 and the remaining PTEs calculated as 1.0 [44,45]. *AT* represents the number of days, d; *BW* is the average body weight, calculated separately for children and adults as 15 kg and 70 kg, respectively.

The hazard quotient (*HQ*) and the hazard index (*HI*) for PTE exposure in children and adults were calculated using the following formulas. If *HQ* and *HI* values were greater than 1, it indicated that PTEs may have adverse effects on health. If *HQ* and *HI* values were less than 1, it suggested that the risks of noncarcinogenic effects on humans are minimal.

(6) $$HQ_{i}=ADI_{i}/RfD_{i}$$

(7) $$HI=\sum_{i=1}^{n}HQ_{i}$$

where *RfDi* is the reference dose for PTE *i*, which can be determined based on the US EPA’s Risk Information System. The reference doses for Cd, Cr, Cu, Zn, Pb, and As are 0.0003, 1.5, 0.04, 0.3, 0.00014, and 0.001, respectively [46].

The sampling map in this study was created using ArcGIS (version 10.2). The data were tested for normality using the Shapiro–Wilk test, and the results indicated that the data followed a normal distribution. Data analysis and data visualization were conducted in Matlab (version R2023a), Sigmaplot (version 14.0), and Microsoft Excel (version 2021).

## 3. Results

### 3.1. Differences in PTE Content among the Five Water Areas with Coal Mining Subsidence

The average content of Cu, Cr, Hg, Pb, Zn, Cd, and As in the sediment of the five stagnant water areas are shown in Table 2. The results indicated that among all the stagnant water areas, Zn had the highest average content, followed by Mn, Zn, Cr, Cu, Pb, As, Cd, and Hg. Comparing the five stagnant water areas, untreated coal mining subsidence water area E had higher contents of Hg, Pb, and Cd compared with areas A, B, C, and D. The highest contents of Cu and Zn were found in the aquaculture pond B.

**Figure 2:**
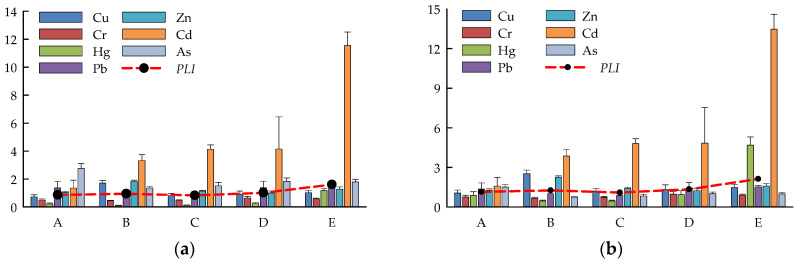
Evaluation index of PTE pollution in sediments. (**a**) with reference to UCG; (**b**) with reference to the background values in Jiangsu Province.

We evaluated the PTE pollution in the soil the study area with reference to the average values of chemical composition in the Upper Continental guidelines (UCG) and sediment quality standards, respectively. In addition, it is also useful to assess the level of PTE pollution in a specific area using the background values of PTEs as standards. The soil PTE background values represent the baseline values of the soil environment in that area that is either unaffected or minimally affected by human activities. The distribution of evaluation indices is shown in Figure 2. Based on the average values of the single-factor pollution index, when referring to UCG, the ranking of single-factor pollution indices for the seven studied PTEs was as follows: Cd (4.90) > As (1.85) > Zn (1.26) > Pb (1.24) > Cu (1.04) > Cr (0.53) > Hg (0.37). When referring to the soil background values in Jiangsu Province, the single-factor pollution index of each PTE followed the pattern Cd (5.72) > Zn (1.56) > Cu (1.53) > Hg (1.49) > Pb (1.24) > As (1.02) > Cr (0.81). In terms of the average values of all sampling points, Cd exhibited the highest pollution level in the sediments, indicating severe contamination.

In terms of the *PLI*, the values of A, B, and C were all below 1 when referring to the UCG, indicating the absence of environmental pollution. However, the *PLI* values of D and E were 1.02 and 1.61, respectively, indicating the presence of some degree of overall PTE pollution. When referring to the background values in Jiangsu Province, all sampling points had *PLI* values higher than 1, indicating the presence of PTE pollution. Among them, sampling point E had the highest *PLI* value, which was 2.13.

With reference to the SQGs, it can be observed that for the sediment of Anguo Landscaped Wetland (A), the level of Cu was below the *TEL*, indicating that adverse biological effects were unlikely to occur. The Cu levels in the sediment of the other four waterlogged areas fell between the *TEL* and *PEL*, suggesting that occasional adverse biological effects may occur.

The Cr levels in the sediment of sampling points A, B, and C were below the *TEL*, which indicated that adverse biological effects were unlikely to occur. However, in the sediment of C and D, the Cr contents were between the *TEL* and *PEL*, suggesting that occasional adverse biological effects may occur. In the sediment of all five waterlogged areas, the levels of Hg, Pb, and Zn were below the *TEL*, indicating that adverse impacts were unlikely to occur. Additionally, the Cd level in all waterlogged areas (except E) was below the *TEL*, suggesting a low likelihood of adverse effects. As for As, the values in the other sampling zones except for B were above the *TEL* but below the *PEL*, indicating that occasional adverse biological effects may occur.

### 3.2. Ecological Risk Assessment

**Figure 3:**
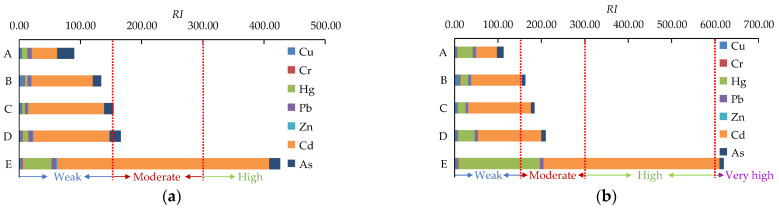
Ecological risk of PTEs in sediment. (**a**) with reference to UCG; (**b**) with reference to the background values in Jiangsu Province.

The ecological risk of PTEs was evaluated with reference to the UCG. The $E_{r}^{i}$ and *RI* are shown in Figure 3a. The $E_{r}^{i}$ values of Cu, Cr, Hg, Pb, Zn, and As were below 40 in all five waterlogged areas, indicating that they were at a weak ecological risk level. The $E_{r}^{i}$ value of Cd in sampling point A fell between 40 and 80, suggesting that Cd was at a moderate ecological risk level. However, the $E_{r}^{i}$ values of Cd in B, C, and D were 99.51, 123.77, and 124.39, respectively, indicating that Cd was at a high ecological risk level. The $E_{r}^{i}$ value of Cd in E was as high as 346.50, indicating that Cd was at an extremely high level of ecological risk in the waterlogged area E with untreated coal mining subsidence. Specifically, the *RI* values of sediments A and B were below 150, indicating a weak ecological risk. The *RI* values of sediments C and D fell between 150 and 300, indicating a moderate ecological risk. The sediment at point E had the highest *RI* value, reaching 426.44, which indicates a high ecological risk level.

The $E_{r}^{i}$ and *RI* of the PTEs in the sediment are shown in Figure 3b, with reference to the soil background values in Jiangsu Province. By comparing the contribution of the $E_{r}^{i}$ of individual PTEs to *RI*, it can be observed that Cd made the highest contribution. In areas A, B, C, D, and E, the contributions of Cd were 42.09%, 71.25%, 78.25%, 69.13%, and 65.18%, respectively. The *RI* values of the five waterlogged areas showed the following pattern: E (620.18) > D (209.93) > C (184.52) > B (162.94) > A (113.14). The results indicated that area E was at a very high ecological risk level, while areas B, C, and D were at a moderate ecological risk level, and area A had a weak ecological risk.

### 3.3. Health Risk Assessment

**Figure 4:**
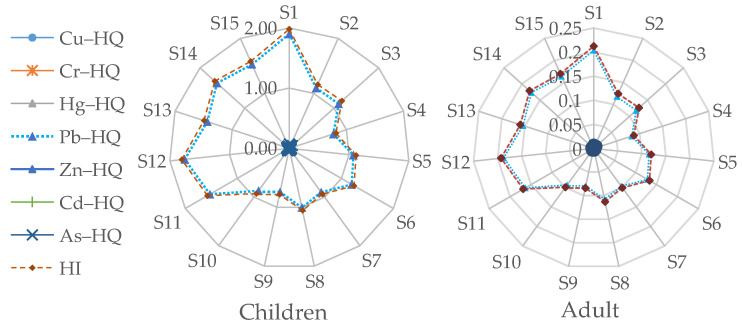
Radar plot for hazard index (*HI*) of each sampling location in the study area for adults and children.

The *HQ* and *HI* values for children and adults for each site are shown in Figure 4. It can be seen that the highest *HQ* and *HI* values for children and adults were observed at sampling point S1 in landscaped wetland A. This was because S1 was the outlet of treated wastewater from a sewage treatment plant, resulting in a high PTE content. After the influence of subsurface flow wetlands, lateral flow wetlands, and other factors, the PTE content at the remaining two sampling points, S2 and S3 in landscaped wetland A, was relatively lower. The three sampling points S13, S14, and S15 in the waterlogged area E with untreated coal mining subsidence all had higher *HQ* and *HI* values.

Table 3 displays the noncarcinogenic risk assessment results for Cu, Cr, Hg, Pb, Zn, Cd, and As in the sediment of each waterlogged area. The noncarcinogenic risk assessment for children indicated that Pb had a *HQ* value higher than the threshold value of 1, indicating a certain noncarcinogenic health risk for children. For other PTEs, the *HQ* values for children were below the threshold value of 1, indicating no noncarcinogenic health risks for children associated with those metals. The *HQ* values of Cu, Cr, Hg, Pb, Zn, Cd, and As for adults were below the threshold value of 1 in all the sampling sites, indicating no noncarcinogenic health risks for adults associated with these PTEs. However, due to the *HQ* value of Pb for children being higher than 1, the *HI* values (excluding C) for all waterlogged areas were also higher than 1 for children. Among them, area E had the highest *HI* value, indicating that it posed the greatest noncarcinogenic risk for children.

### 3.4. Variation Trends of Soil PTEs at Different Distances

**Figure 5:**
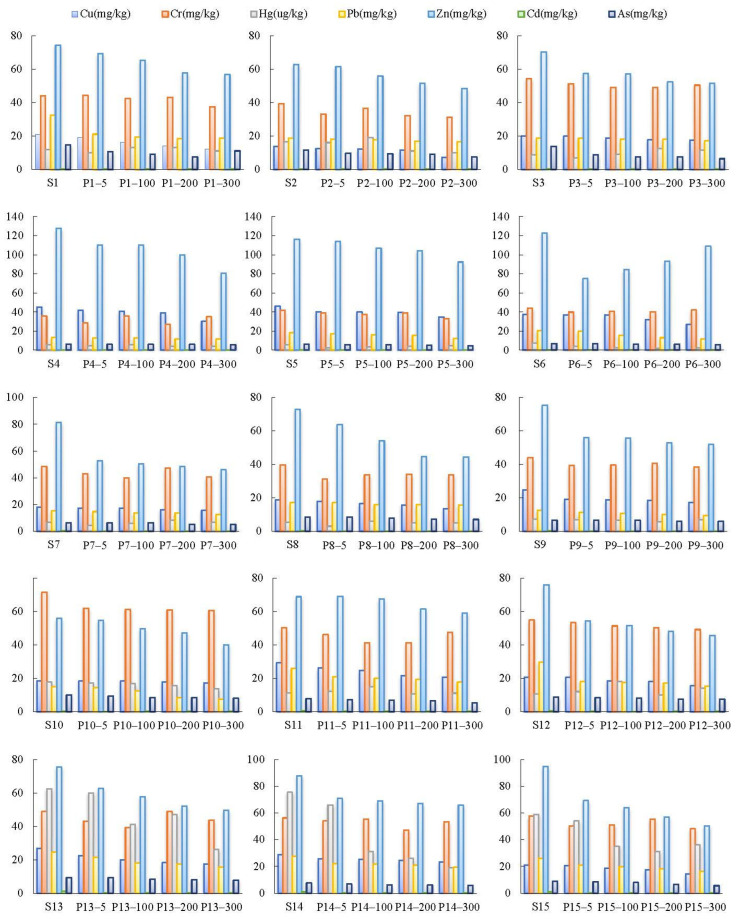
Variation trends of PTE content at each sampling point (distance from the waterlogged areas: 5 m, 100 m, 200 m, 300 m).

The main source of PTEs in the sediment from waterlogged areas with coal mining subsidence is human activities, including mining activities and the use of pesticides and fertilizers [49]. Due to the lower topography of the waterlogged areas with coal mining subsidence, PTEs from surrounding farmland soils enter these areas through surface runoff. In theory, the closer the distance to the waterlogged areas with coal mining subsidence, the higher the soil’s PTE content. Therefore, the sediment in waterlogged areas with coal mining subsidence is expected to have a higher content of PTEs compared with the region overall. In this study, the variation trends of the PTE content in soil surrounding the waterlogged areas with coal mining subsidence are shown in Figure 5. It can be found that the contents of Cu, Pb, Zn, and As increase as the distance from the waterlogged areas decreases, which was in line with theoretical expectations. However, there was no clear pattern observed in the variation in the Cr, Hg, and Cd content in relation to the distance in the soil.

### 3.5. Analysis of the Correlation between PTE Content and Distance, SOC, and pH

**Figure 6:**
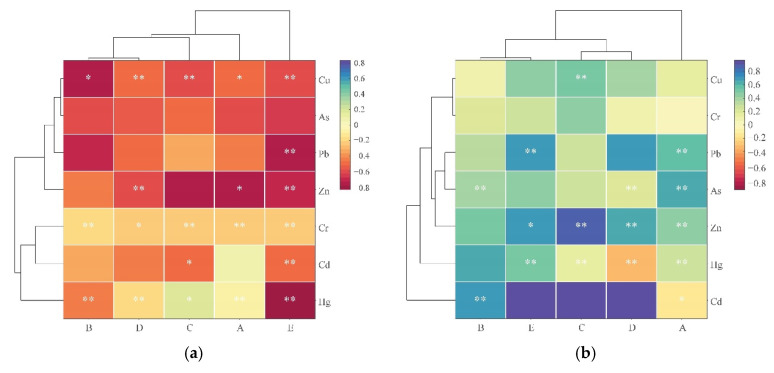

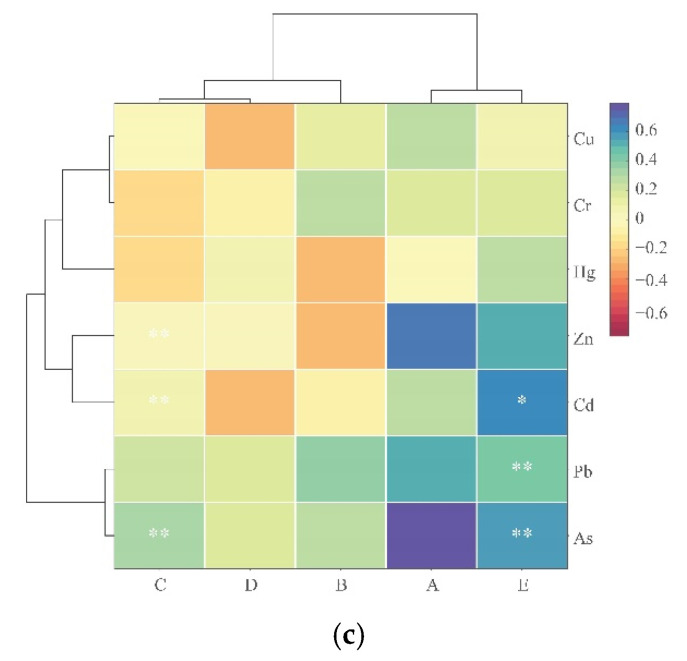
Pearson correlation analysis of PTE contents and distance, SOC, and pH (** represents significance at the 0.05 level, * represents significance at the 0.1 level). (**a**) Distance; (**b**) SOC; (**c**) pH.

The enrichment and migration of PTEs are influenced by the distance, SOC, and pH. To analyze their correlations, we used the Pearson correlation coefficient method to analyze the correlations between PTEs and the distance, SOC, and pH. The results of the analysis are shown in Figure 6. From Figure 6a, it can be observed that in Study Area A, Cu and Zn were significantly negatively correlated with distance (*p* < 0.1), while Cr and Hg were significantly negatively correlated with distance (*p* < 0.05). In Study Area B, Cu was significantly negatively correlated with distance (*p* < 0.1), and Cr and Hg were significantly negatively correlated with distance (*p* < 0.05). In Study Area C, Cu and Cr were significantly negatively correlated with distance (*p* < 0.05), and Cd was significantly negatively correlated with distance (*p* < 0.1). In Study Area D, Cu, Zn, and Hg were significantly negatively correlated with distance (*p* < 0.05). In Study Area E, Cu, Pb, Zn, Cr, Cd, and Hg were significantly negatively correlated with distance (*p* < 0.05).

As shown in Figure 6b, in Study Area A, Pb, As, Zn, and Hg were significantly positively correlated with SOC (*p* < 0.05). In Study Area B, As and Cd were significantly positively correlated with SOC at the 0.05 significance level. In Study Area C, Cu and Zn were significantly positively correlated with SOC (*p* < 0.05). In Study Area D, As and Zn were significantly positively correlated with SOC at the 0.05 significance level. In Study Area E, Pb and Hg were significantly positively correlated with SOC (*p* < 0.05). As shown in Figure 6c, in Study Area C, As was significantly positively correlated with pH (*p* < 0.05). In Study Area E, Pb and As were significantly positively correlated with pH (*p* < 0.05), and Cd was significantly positively correlated with pH at the 0.1 significance level.

**Figure 7:**
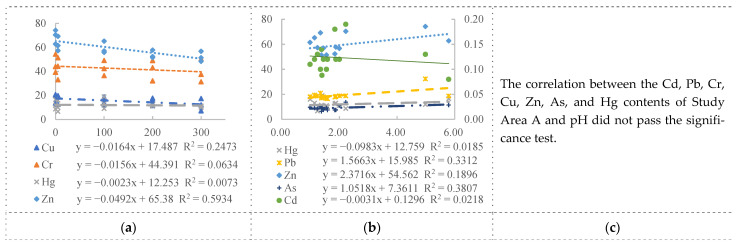

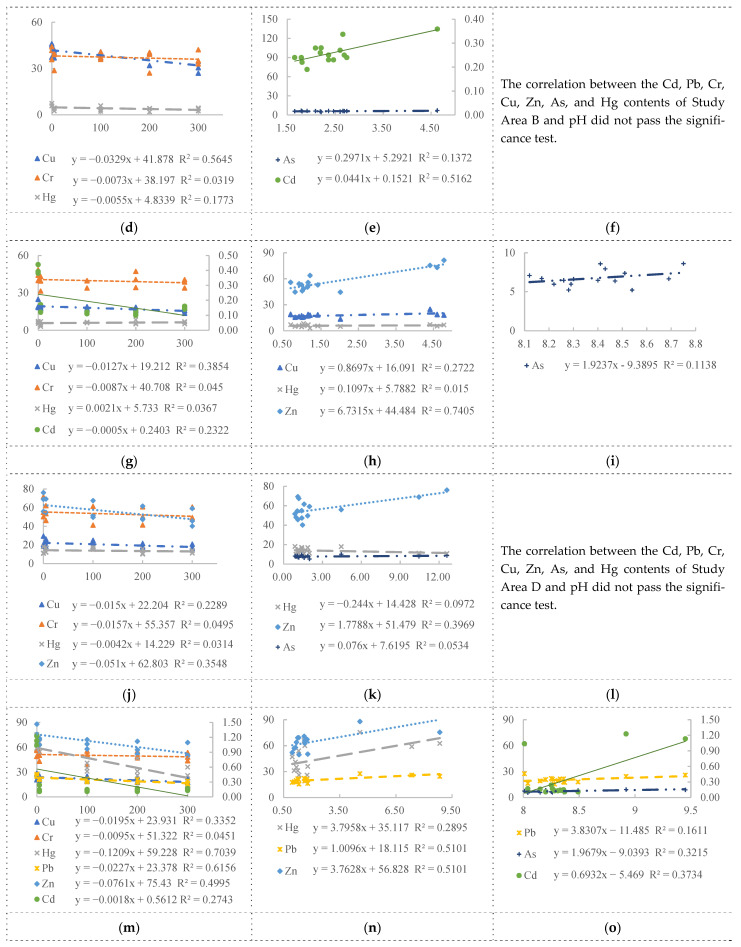
Linear correlation between PTEs and distance, SOC, and pH. PTEs without any significant correlation have been omitted as unnecessary. (**a**) Linear correlation between PTEs and distance in area A; (**b**) Linear correlation between PTEs and SOC in area A; (**c**) Linear correlation between PTEs and pH in area A; (**d**) Linear correlation between PTEs and distance in area B; (**e**) Linear correlation between PTEs and SOC in area B; (**f**) Linear correlation between PTEs and pH in area B; (**g**) Linear correlation between PTEs and distance in area C; (**h**) Linear correlation between PTEs and SOC in area C; (**i**) Linear correlation between PTEs and pH in area C; (**j**) Linear correlation between PTEs and distance in area D; (**k**) Linear correlation between PTEs and SOC in area D; (**l**) Linear correlation between PTEs and pH in area D; (**m**) Linear correlation between PTEs and distance in area E; (**n**) Linear correlation between PTEs and SOC in area E; (**o**) Linear correlation between PTEs and pH in area E.

To further analyze the relationship between PTEs and the distance, soil SOC, and pH in the surrounding soil of water areas with coal mining subsidence, we conducted a linear correlation analysis of PTEs with the distance, SOC, and pH using significant correlation tests. The results are shown in Figure 7. In Study Area A, the explanatory power of the distance on the Cu and Zn content was 0.2473 and 0.5934, respectively. The explanatory power of the SOC on the Pb and As content was 0.3312 and 0.3807, respectively. In Study Area B, the explanatory power of the distance on the Cu content was 0.5645, and the explanatory power of the SOC on the Cd content was 0.5162. In Study Area C, the explanatory power of the distance on the Cu content was 0.3854, and the explanatory power of the SOC on the Zn content was 0.7405. In Study Area D, the explanatory power of the distance on the Zn content was 0.3548, and the explanatory power of the SOC on the Zn content was 0.3969. In Study Area E, the explanatory power of the distance on the Cu, Hg, Pb, and Zn content was 0.3352, 0.7039, 0.6165, and 0.4995, respectively. The explanatory power of the SOC on the Pb and Zn content was 0.5101 for both. Overall, the distance had a relatively high explanatory power for Cu and Zn, and a relatively low explanatory power for Cr and Hg.

## 4. Discussion

### 4.1. Developing as Landscaped Wetlands, Fish Farming, Fish–Photovoltaic Complementary Wetland, and Photovoltaic Wetlands Can All Help Reduce the Comprehensive PTE Pollution in Waterlogged Areas with Coal Mining Subsidence

By comparing five waterlogged areas with coal mining subsidence, it can be observed that the contents of Hg, Pb, and Cd in the sediments of landscaped wetland A, fish farming wetland B, fish–photovoltaic complementary wetland C, and photovoltaic wetland D were lower than those in the untreated waterlogged area E. The *PLI* results also indicated that areas A, B, and C did not have PTE pollution, while D exhibited a certain degree of comprehensive PTE pollution, but significantly lower than the untreated waterlogged area E with coal mining subsidence. The *RI* values of the five waterlogged areas followed the pattern E > D > C > B > A.

Wetland landscapes were connected to other surface water bodies, which enhanced the self-purification capacity of the water in waterlogged areas. The content of PTEs in sediments can be reduced through the comprehensive effects of physical, chemical, biological, and other processes. In addition, waterlogged areas with landscaped wetlands were often planted with aquatic plants such as reeds and cattails, which had the ability capability to remove PTEs [50,51,52]. This It can help mitigate the level of PTE pollution in aquatic ecosystems to a certain extent.

The main reason for the reduction in PTE content in sediments was the transfer of PTEs in the aquatic food web in fish farming and fish–photovoltaic complementary wetlands [53,54]. It is worth mentioning that aquaculture feeds had relatively high contents of Cu and Zn, which can introduce certain amounts of Cu and Zn into the system. However, compared with other PTEs, Cu and Zn led to lower ecological and health risks. Lastly, photovoltaic wetlands integrated solar photovoltaic power generation with wetland ecosystems, providing the dual benefits of water purification and electricity generation. Through these development approaches, it is possible to comprehensively reduce the PTE pollution level in waterlogged areas with coal mining subsidence and achieve sustainable resource utilization and environmental protection.

### 4.2. Cu Showed Clear Diffusion Patterns

The process of PTE migration was complex and influenced by various factors, including the characteristics of the PTEs themselves and environmental factors [55]. During the migration of PTEs from soil to waterlogged areas, physical, chemical, and biological interactions occurred between the PTEs and the soil’s active components [8,9,10]. For example, PTEs can be adsorbed by soil colloids, undergo ion exchange with soil solutions, or be absorbed by vegetation. Relevant studies have shown that the metal content generally decreased with distance [56,57]. The findings of this study were similar to the results obtained by Zhengxi Gao [58], where Cu exhibited clear diffusion patterns, with higher soil PTE content observed closer to waterlogged areas. The low-lying topography of the waterlogged area leads to the entry of PTEs into the soil through factors such as rainwater runoff. The smaller the distance to the waterlogged area, the higher the accumulation of PTEs in the soil.

Additionally, the SOC also played an important role in the adsorption and migration of PTEs in the soil. In this study, there was a strong correlation between Cd and the SOC. The SOC can affect the adsorption of Cd, reducing the activity of Cd ions. It can also form complexes or chelates with Cd, altering the speciation of Cd and influencing its migration and transformation. In future studies, researchers can provide clearer explanations for their migration patterns by analyzing the state of PTE occurrence.

## 5. Conclusions

This paper investigated the migration patterns and influencing factors of soil from coal mining subsidence areas to waterlogged areas under different remediation modes for the first time. The PTE content, such as Cu, in the soil increased with proximity to the waterlogged area. The migration patterns were influenced by SOC and pH. Landscape development, aquaculture, development of fish–photovoltaic complementary wetland, and photovoltaic wetland development can all reduce the overall PTE pollution in waterlogged areas with coal mining subsidence. Currently, large-scale restoration is urgently needed in waterlogged areas with coal mining subsidence, and these research findings can provide data support for the selection of restoration modes and effective control of heavy metals in such areas.

## Figures and Tables

**Table 1:** Classification of $E_{r}^{i}$ and *RI*.

$E_{r}^{i}$	*RI*	Ecological Risk Level
$E_{r}^{i}$ ≤ 40	*RI* ≤ 150	Low
40 < $E_{r}^{i}$ ≤ 80	150 < *RI* ≤ 300	Moderate
80 < $E_{r}^{i}$ ≤ 160	300 < *RI* ≤ 600	High
160 < $E_{r}^{i}$ ≤ 320	600 ≤ *RI*	Very high
320 < $E_{r}^{i}$		Extremely high

**Table 2:** Content of PTEs in sediments.

	Cu	Cr	Hg	Pb	Zn	Cd	As
	mg kg^−1^	mg kg^−1^	ug kg^−1^	mg kg^−1^	mg kg^−1^	mg kg^−1^	mg kg^−1^
A	18.09 ± 3.87	45.88 ± 7.7	12.35 ± 3.96	23.3 ± 7.88	69.12 ± 5.85	0.13 ± 0.06	13.27 ± 1.69
B	42.82 ± 4.83	40.53 ± 4.26	6.33 ± 0.95	17.27 ± 3.74	122.45 ± 5.77	0.33 ± 0.04	6.49 ± 0.48
C	20.57 ± 3.63	44.16 ± 4.34	6.45 ± 0.9	15.04 ± 2.27	76.6 ± 4.33	0.4 ± 0.03	7.26 ± 1.18
D	22.82 ± 5.76	58.87 ± 11.22	13.24 ± 4	23.55 ± 7.77	66.92 ± 10.22	0.41 ± 0.23	8.52 ± 1.17
E	25.39 ± 4.11	54.29 ± 4.65	65.64 ± 8.67	26.02 ± 1.57	86.09 ± 9.75	1.13 ± 0.09	8.57 ± 0.93
UCG [47,48]	25	92	56	17	67	0.098	4.8
Background values	17	60	14	17	54	0.084	8.7
*TEL*	18.7	52.3	170	30.2	124	0.68	7.2
*PEL*	108.2	160.4	170	112.2	271	4.2	41.6

UCG: upper continental guidelines, *TEL*: threshold effect level, *PEL*: probable effect level.

**Table 3:** Health risk assessment results for PTEs in sediment under different restoration modes in waterlogged areas with coal mining subsidence.

		Cu*-HQ*	Cr*-HQ*	Hg*-HQ*	Pb*-HQ*	Zn*-HQ*	Cd*-HQ*	As*-HQ*	*HI*
**Children**									
A	mean	3.72 × 10^−^^3^	2.51 × 10^−4^	6.34 × 10^−4^	1.37	1.89 × 10^−3^	3.65 × 10^−5^	6.54 × 10^−2^	1.44
	sd	7.95 × 10^−4^	4.22 × 10^−5^	2.03 × 10^−4^	4.63 × 10^−1^	1.60 × 10^−4^	1.51 × 10^−5^	8.34 × 10^−3^	0.00
B	mean	8.80 × 10^−3^	2.22 × 10^−4^	3.25 × 10^−4^	1.01	3.35 × 10^−3^	8.91 × 10^−5^	3.20 × 10^−2^	1.06
	sd	9.92 × 10^−4^	2.34 × 10^−5^	4.87 × 10^−5^	2.19 × 10^−1^	1.58 × 10^−4^	1.12 × 10^−5^	2.36 × 10^−3^	0
C	mean	4.23 × 10^−3^	2.42 × 10^−4^	3.31 × 10^−4^	8.83 × 10^−1^	2.10 × 10^−3^	1.11 × 10^−4^	3.58 × 10^−2^	9.26 × 10^−1^
	sd	7.46 × 10^−4^	2.38 × 10^−5^	4.63 × 10^−5^	1.33 × 10^−1^	1.19 × 10^−4^	8.42 × 10^−6^	5.81 × 10^−3^	0.00
D	mean	4.69 × 10^−3^	3.23 × 10^−4^	6.80 × 10^−4^	1.38	1.83 × 10^−3^	1.11 × 10^−4^	4.35 × 10^−2^	1.43
	sd	1.18 × 10^−3^	6.15 × 10^−5^	2.06 × 10^−4^	4.56 × 10^−1^	2.80 × 10^−4^	6.17 × 10^−5^	5.77 × 10^−3^	0.00
E	mean	5.22 × 10^−3^	2.97 × 10^−4^	3.37 × 10^−3^	1.53	2.36 × 10^−3^	3.10 × 10^−4^	4.23 × 10^−2^	1.58
	sd	8.44 × 10^−4^	2.55 × 10^−5^	4.45 × 10^−4^	9.19 × 10^−2^	2.67 × 10^−4^	2.60 × 10^−5^	4.57 × 10^−3^	0.00
**Adults**									
A	mean	3.98 × 10^−4^	2.69 × 10^−5^	6.80 × 10^−5^	1.47 × 10^−1^	2.03 × 10^−4^	3.91 × 10^−6^	7.01 × 10^−3^	1.54 × 10^−1^
	sd	8.52 × 10^−5^	4.52 × 10^−6^	2.18 × 10^−5^	4.96 × 10^−2^	1.72 × 10^−5^	1.62 × 10^−6^	8.94 × 10^−4^	0.00
B	mean	9.43 × 10^−4^	2.38 × 10^−5^	3.49 × 10^−5^	1.09 × 10^−1^	3.59 × 10^−4^	9.54 × 10^−6^	3.43 × 10^−3^	1.13 × 10^−1^
	sd	1.06 × 10^−4^	2.50 × 10^−6^	5.22 × 10^−6^	2.35 × 10^−2^	1.69 × 10^−5^	1.20 × 10^−6^	2.52 × 10^−4^	0.00
C	mean	4.53 × 10^−4^	2.59 × 10^−5^	3.55 × 10^−5^	9.46 × 10^−2^	2.25 × 10^−4^	1.19 × 10^−5^	3.83 × 10^−3^	9.92 × 10^−2^
	sd	7.99 × 10^−5^	2.55 × 10^−6^	4.96 × 10^−6^	1.43 × 10^−2^	1.27 × 10^−5^	9.02 × 10^−7^	6.22 × 10^−4^	0.00
D	mean	5.02 × 10^−4^	3.46 × 10^−5^	7.29 × 10^−5^	1.48 × 10^−1^	1.96 × 10^−4^	1.19 × 10^−5^	4.66 × 10^−3^	1.54 × 10^−1^
	sd	1.27 × 10^−4^	6.59 × 10^−6^	2.20 × 10^−5^	4.89 × 10^−2^	3.00 × 10^−5^	6.61 × 10^−6^	6.18 × 10^−4^	0.00
E	mean	5.59 × 10^−4^	3.19 × 10^−5^	3.61 × 10^−4^	1.64 × 10^−1^	2.53 × 10^−4^	3.32 × 10^−5^	4.53 × 10^−3^	1.69 × 10^−1^
	sd	9.05 × 10^−5^	2.73 × 10^−6^	2.77 × 10^−5^	9.84 × 10^−3^	2.86 × 10^−5^	2.78 × 10^−6^	4.89 × 10^−4^	0.00

sd represents the standard deviation of the data.

## Data Availability

Not applicable.
